# Laparoscopic Radical Prostatectomy: The Learning Curve of a Low Volume Surgeon

**DOI:** 10.1155/2013/974276

**Published:** 2013-03-03

**Authors:** Anuar I. Mitre, Mario F. Chammas, José Eugênio A. Rocha, Ricardo Jordão Duarte, Gustavo Xavier Ebaid, Flavio Trigo Rocha

**Affiliations:** ^1^Division of Urology, University of São Paulo, 05403 Sao Paulo, SP, Brazil; ^2^Urology Section, Hospital Sirio Libanes, 05403 Sao Paulo, SP, Brazil

## Abstract

*Objective*. Analyze the learning curve for laparoscopic radical prostatectomy in a low volume program. *Materials and Methods*. A single surgeon operated on 165 patients. Patients were consecutively divided in 3 groups of 55 patients (groups A, B, and C). An enhancement of estimated blood loss, surgery length, and presence of a positive surgical margin were all considered as a function of surgeon's experience. *Results*. Operative time was 267 minutes for group A, 230 minutes for group B, and 159 minutes for group C, and the operative time decreased over time, but a significant difference was present only between groups A and C (*P* < 0.001). Mean estimated blood loss was 328 mL, 254 mL, and 206 mL (*P* = 0.24). A conversion to open surgery was necessary in 4 patients in group A. Positive surgical margin rates were 29.1%, 21.8%, and 5.5% (*P* = 0.02). Eight patients in group A, 4 patients in group B, and one in group C had biochemical recurrence. *Conclusion*. Significantly less intraoperative complications were evident after the first 51 cases. All other parameters (blood loss, operative time, and positive surgical margins) significantly decreased and stabilized after 110 cases. Those outcomes were somehow similar to previous published series by high-volume centers.

## 1. Introduction

Introduction of the laparoscopic approach has revolutionized the field of minimal invasive surgery, and in the modern era laparoscopic radical prostatectomy (LRP) has been described as a standard and reproducible surgical procedure in many centers worldwide [1].

In the same way of other modern surgical techniques, laparoscopic procedures necessitate new training methods and an amount of surgical procedures performed before a surgeon reaches an accepted stage of expertise in outcome parameters. The length of this learning curve is proportional to the complexity of the procedure. Several authors have published their results, and some learning curves, about laparoscopic radical prostatectomy, considered a highly complex surgery [2–4].

However, most of these studies have been conducted in large academic centers with a high volume of radical prostatectomies. Whether those same learning curves are reproducible in low volume centers by less experienced surgeons remains an unanswered question. Therefore, in this paper, we aimed to analyze the learning curve for extraperitoneal laparoscopic radical prostatectomy in a low volume environment.

## 2. Materials and Methods

Between August 2003 and June 2011, we have performed 165 LRP procedures. The study had been authorized by the appropriate ethics committee, and informed consents obtained. Patients were ordered chronologically from number 1 to number 165 for this study. The patients were divided into 3 groups for prospective analysis: group 1 consisted of the first 55 patients, group 2 was formed by the patients numbered from 56 to 110, and the final 55 patients were included in the group 3. 

All surgeries were performed by a single surgeon (AIM), and all patients were diagnosed previously as localized prostatic adenocarcinoma. All procedures were performed using the extraperitoneal surgical technique using five trocars [5]. Antegrade dissection was done, and the prostate pedicles controlled with polymer clips (Hem-o-lock, Weck Closure Systems, Research Triangle. Park NC, USA). Urethrovesical anastomosis was performed with a running suture, and lymphadenectomy performed in the presence of a PSA higher than 20 ng/dL, Gleason 7 (4 + 3) or higher, or presence of suspicious nodes described in preoperative imaging studies. 

Patients were then treated following the local hospital clinical pathway of LRP, and postoperative analgesia included administration of nonsteroidal anti-inflammatory drugs intravenously during the first 24 h and orally thereafter until discharge. On the first postoperative day physical therapists assisted in mobilization, and patients were reefed the evening of the day of surgery. All radical prostatectomy specimens were evaluated by a specialized uropathologist.

The length of surgery, intraoperative blood loss, transfusion rates, intra- and postoperative complications, conversion, histopathologic results, and oncological outcome (PSA recurrence) were determined in all groups and compared. All patients were discharged with a temporary bladder catheter in place, which was typically removed on the seventh day after surgery. Complications were classified according to the 2004 Clavien-Dindo classification [6].

Postoperative erectile dysfunction and incontinence were not evaluated in this paper. A positive surgical margin was defined as the presence of tumour cells in contact with the inked surface of the specimen. 

In order to establish the learning curve, all variables were calculated and recorded, and a comparison between the groups was performed. Statistical analysis was performed with the SPSS program (Statistical Package for Social Sciences, version 11.01, Chicago, IL). For qualitative variables the absolute (*n*) and relative (%) frequencies were recorded. The chi-square analysis was used to compare those variables among them. Significance was established at *P* < 0.05.

ANOVA was used to compare variables with continuous values. In order to evaluate the equality of group variances the Brown-Forsythe test was also applied. In the presence of significant differences between the groups, comparisons were then performed using the Bonferroni test. 

## 3. Results

For the 165 patients, we observed a median age of 61.7 (44–83) years, a median prostate size of 39.07 g (15–150 g), and a median preoperative PSA of 6.66 ng/mL (1.8–39.9); those values were equivalent in all groups (*P* > 0.05). 

The parameters found in groups A, B, and C, respectively, were the following: mean surgical time: 267.1 (±64.3), 230 (±65.0), and 159.5 (±35.5) min (*P* < 0.001). Loss of blood was 328 (±188), 254 (±129), and 206 (±95) mL, and a significant difference was present only between groups A and C (*P* < 0.001). Eleven patients (20.0%) in group A and one (1.8%) in group B required blood transfusion. No transfusions were necessary in group C (*P* = 0.010).

Prolonged urine leakage was observed in seven patients (12.7%) in group A and in seven patients (12.7%) in group B. 

Rectal lesions occurred in 3 patients (5.4%) in group A; the injuries were repaired intraoperatively with polyglactin 3–0 sutures in two planes. Another patient in group A sustained an intraoperative bladder injury and also repaired laparoscopically. No intraoperative lesions were recorded in groups B or C. 

An analysis of postoperative complications (Clavien-Dindo classification) showed a significant difference between the groups (*P* < 0.05). Clavien I and III were more frequent in group B and II in group A. Two patients in group C presented postoperative issues (Table 1).

Conversion to open surgery was deemed necessary in 4 patients (7.3%) in group A. No other conversions were then required. 

In groups A, B and C, margins were respectively positive on: 29.1% (16), 21.8% (12) and 5.5% (3) patients. When positive margins were correlated to the clinical stage of the disease a higher incidence was demonstrated in pT3a and pT3b individuals when compared to other patients (Table 2). 

Due to a high risk of local relapse, 13.9% (23) of patients received radiotherapy.

After a minimum followup of 20 months (20–97 months), 8 patients in group A, 4 patients in group B, and one patient in group C had biochemical recurrence (PSA < 0.2 ng/mL).

## 4. Discussion 

Historically, open radical prostatectomy is the standard surgical treatment for localized prostate cancer in patients in good health [10]. However, this procedure was not widely accepted until 1982 when a refined and reproducible method was described by Walsh and Donker [11]. Sixteen years later a laparoscopic technique for the management of localized prostate cancer was suggested by Schuessler et al, but the conclusions learned from the initial series were that this was a lengthy and difficult procedure, and little advantage was added compared to the open counterpart [12].

The initial procedure was revised [13] and over the latest years, the laparoscopic technique has shown substantial efficacy [4]. The benefits of the minimally invasive approach were reported in several series, but until recently, the procedure was limited to specialized centers, mainly due to a steep learning curve.

This difficulty is attributed to the counter-intuitive motion, two-dimensional visualization, and lack of articulating instruments for standard laparoscopic surgery. Several authors have evaluated their initial series, and some learning curves were proposed, mainly in academic centers with high surgery volume [8, 14, 15]. 

On the other hand, even after reviewing large series it seems it is not yet possible to estimate the number of cases required for a novice surgeon to master the skills necessary to perform a laparoscopic radical prostatectomy. In a review of their first 1311 cases Vallancien et al. [16] suggested that at least 50 difficult operations, with at least one case/week during the first year, were required to master complex laparoscopic urological procedures. Conversely, the records from 8,544 consecutive patients with prostate cancer treated laparoscopically by 51 surgeons at 14 academic institutions in Europe and the USA were evaluated in a multicenter study evaluating the presence of positive surgical margins as an effect of the surgeons' experience, and an apparent improvement in surgical margin rates up to a plateau was demonstrated only after 200 to 250 surgeries [16]. 

As seen in previous reports, a drop in the complications rate was demonstrated as the surgeon's experience increased. In our experience significantly less intraoperative complications and conversions to open surgery were evident after the first 51 cases, and blood loss and operative time continued to significantly decrease and stabilized after 110 cases. On a study published by Starling et al. [17] an improvement of the surgical and functional parameters occurred after 70 cases. On the other hand, in series of high-volume academic centers residents without prior experience required 38–52 cases to be considered competent [7, 18].

This same evolution was present when oncological parameters were evaluated. In our series the positive surgical margins also significantly decrease and stabilized after 110 cases. Our overall incidence of positive surgical margins was 20%, and this was similar to the series presented by Katz et al. [19]. As expected, the incidence of positive margins in our series increased in pT3 tumors and varies in pT2 tumors among the series; this was also present when other authors presented their learning curves [19]. In a study published by Bollens et al. 11 of 50 patients presented positive surgical margins after laparoscopic prostatectomy, and of these 11 cases two were pT2 tumors [20]. Rassweiler et al. reported, after reviewing 180 patients, a 16% incidence of positive, and almost half of patients had pT3 tumors [21]. Guillonneau and Vallancien presented a 14% incidence of positive margins in pT2b tumors and 33% in pT3a [22].

In our study no patient had biochemical recurrence after 110 cases. In a study published by Vickers et al. the probability of recurrence initially dropped steeply then reached a plateau after 250–350 surgeries [23]. Similarly, a large multicenter study demonstrated a plateau also at 200 to 250 surgeries [9].

Interestingly, this same retrospective study conducted by Vickers et al. suggested that 750 laparoscopic radical prostatectomies were necessary in order to reach results equivalent to the open approach [23]. 

Our series was performed by a single surgeon with previous laparoscopic experience with other kinds of surgery but no direct supervision during the learning curve. Other authors suggested that the learning curve could have been shortened if a training program under supervision has been used. One proposed method is the Leipzig model, where an expert mentor acts as a first assistant, while the student performs the steps corresponding to his or her level, then the student remain as a first assistant for the remainder of the surgery, leading to a gradual learning process [24]. 

The selection between an extraperitoneal and a transperitoneal approaches to the laparoscopic radical prostatectomy depends nowadays mainly on the surgeon's preference. Initially, a transperitoneal approach has been elected as the main access for this procedure, along with an antegrade technique [13], which was reproduced by other centers [22]. Later, Rassweiler et al. described the feasibility of LRP done through the Retzius space, performing the surgery in an retrograde manner and accessing the seminal vesicles after transecting the posterior bladder neck (Heilbronn technique) [21]. An extraperitoneal approach was first described by Raboy et al. [25], following the principles of the French technique and transecting the venous complex and urethra as the final portion of the prostate dissection. 

In our series an extraperitoneal antegrade laparoscopic technique, performed in a similar manner as previously described by Dubernard et al. [5], was used, aiming to unite the advantages of minimally invasive techniques with those of extraperitoneal cavity surgery, also avoiding the extreme Trendelenburg position used in the transperitoneal technique, since the peritoneum retracts the intestines, allowing a more neutral position. Furthermore, this approach may also be suitable for patients with multiple previous abdominal surgeries and in the presence of obesity [26]. 

Our study has several limitations. Initially, only the total operative time was recorded, instead of timing separately each step of the procedure, as done by Dev et al. [3], what could have added substantial information regarding the difficulty and complexity of each step to surpass the initial learning curve. Furthermore, although we have added the biochemical recurrence as one of the parameters, we are aware that our relative short followup may limit the usage of this information in our study. Finally, continence and erectile dysfunctions were not evaluated in our series due to a lack of standardization for urinary incontinence and for the no application of validated questionnaires for erectile dysfunction, but we are aware that those parameters would add important information regarding the functional outcomes for the studied population. 

Finally, it must be noted that in the modern era a laparoscopic robotic approach has been favored over pure laparoscopic surgeries mainly because of its apparent reduced learning curve [27, 28], but, especially in developing countries, its high costs, availability, and the training facilities required are still major issues that are somehow difficult to surmount, particularly in limited budget situations. Therefore, apparently, there is still a role for laparoscopic radical prostatectomy in our days, and studying the learning process and identifying a proper learning curve for this procedure in low-volume centers seem necessary even in the robotics epoch. Additionally, the surgeon with experience in laparoscopic radical prostatectomy may continue by minimally invasive surgery in cases of breakdown of the robot during the surgery avoiding conversion to open procedure and may facilitate the beginning of robotic assisted laparoscopic radical prostatectomy.

## 5. Conclusion

Although further studies seem necessary to unify and identify the number of cases required to master this technique considerable less intraoperative complications and conversions to open surgery were noted after the first 51 cases, and all other parameters (blood loss, operative time, and positive surgical margins) significantly decreased and stabilized after 110 cases in our study. The learning curve for extraperitoneal laparoscopic radical prostatectomy appears to be continuous and the implementation of a successful program was possible even in the presence of a low volume and in the absence of a specific mentorship program in the early learning curve. 

## Figures and Tables

**Table 1:** Postoperative complications (Clavien-Dindo classification).

Complication/Clavien	A		B		C		*P* value
	*n*	%	*n*	%	*n*	%	
Urinary extravasation, temporary elevated creatinine/I	8	14.5%	8	14.5%	1	1.8%	0.001
Blood transfusion/II	11	20%	1	1.8%	0	0%	
Hem-o-lock clip migration to bladder (cystoscopy under local anesthesia)/IIIa	0	0%	0	0%	1	1.8%	
Management of urinoma or infected lymphocele, embolization and colostomy (intervention under general anesthesia)/IIIb	3	5.4%	4	7.3%	0	0%	
Acute pulmonary edema/IV	1	1.8%	0	0.0%	0	0%	

**Table 2:** Positive margins versus stage of the disease.

TNM	Group A		Group B		Group C		total	
	M+/total	%	M+/Total	%	M+/Total	%	M+	%
pT1	0/2	0	0/2	0	0/2	0	0	0
pT2a	0/6	0	1/10	10.0	0/16	0	1	3.8
pT2b	0/4	0	0/1	0	1/7	14.3	1	12.5
pT2c	4/24	16.7	9/35	25.7	1/23	4.3	14	17.1
pT3a	9/14	64.3	2/5	40.0	1/5	20.0	12	50.0
pT3b	3/4	75.0	0/2	0	0/1	0	3	42.8
pT3c	0/1	0	0/0	0	0/0	0	0	0
pT4	0/0	0	0/0	0	0/1	0	0	0

Total	16/55	29.1	12/55	21.8	3/55	5.5	31	18.8

M+: positive margin.

## References

[1] Sandhu GS, Nepple KG, Tanagho YS, Andriole GL (2013). Laparoscopic prostatectomy for prostate cancer: continued role in urology. *Surgical Oncology Clinics of North America*.

[2] Chang SL, Gonzalgo ML (2009). Surgery: laparoscopic prostatectomy: learning curve and cancer control. *Nature Reviews Urology*.

[3] Dev H, Sharma NL, Dawson SN (2012). Detailed analysis of operating time learning curves in robotic prostatectomy by a novice surgeon. *BJU International*.

[4] Hruza M, Weiß HO, Pini G (2010). Complications in 2200 consecutive laparoscopic radical prostatectomies: standardised evaluation and analysis of learning curves. *European Urology*.

[5] Dubernard P, Benchetrit S, Chaffange P, Hamza T, Van Box Som P, Hoznek A (2003). Retrograde extraperitoneal laparoscopic prostatectomy (R.E.L.P.) simplified technique (based on a series of 143 cases). *Progres en Urologie*.

[6] Dindo D, Demartines N, Clavien PA (2004). Classification of surgical complications: a new proposal with evaluation in a cohort of 6336 patients and results of a survey. *Annals of Surgery*.

[7] Stolzenburg JU, Rabenalt R, Do M, Horn LC, Liatsikos EN (2006). Modular training for residents with no prior experience with open pelvic surgery in endoscopic extraperitoneal radical prostatectomy. *European Urology*.

[8] Rassweiler J, Schulze M, Teber D (2005). Laparoscopic radical prostatectomy with the heilbronn technique: oncological results in the first 500 patients. *Journal of Urology*.

[9] Secin FP, Savage C, Abbou C (2010). The learning curve for laparoscopic radical prostatectomy: an international multicenter study. *Journal of Urology*.

[10] Catalona WJ, Ramos CG, Carvalhal GF (1999). Contemporary results of anatomic radical prostatectomy. *CA: A Cancer Journal for Clinicians*.

[11] Walsh PC, Donker PJ (1982). Impotence following radical prostatectomy: insight into etiology and prevention. *Journal of Urology*.

[12] Schuessler WW, Schulam PG, Clayman RV, Kavoussi LR (1997). Laparoscopic radical prostatectomy: initial short-term experience. *Urology*.

[13] Guillonneau B, Cathelineau X, Barret E, Rozet F, Vallancien G (1999). Laparoscopic radical prostatectomy: technical and early oncological assessment of 40 operations. *European Urology*.

[14] Trabulsi EJ, Guillonneau B (2005). Laparoscopic radical prostatectomy. *Journal of Urology*.

[15] Bollens R, Sandhu S, Roumeguere T, Quackels T, Schulman C (2005). Laparoscopic radical prostatectomy: the learning curve. *Current Opinion in Urology*.

[16] Vallancien G, Cathelineau X, Baumert H, Doublet JD, Guillonneau B (2002). Complications of transperitoneal laparoscopic surgery in urology: review of 1,311 procedures at a single center. *Journal of Urology*.

[17] Starling ES, Reis LO, Vaz Juliano R (2010). Extraperitoneal endoscopic radical prostatectomy: how steep is the learning curve? Overheads on the personal evolution technique in 5-years experience. *Actas Urologicas Espanolas*.

[18] Stolzenburg JU, Rabenalt R, Do M, Jiménez Cruz F, Liatsikos EN (2006). Laparoscopic extraperitoneal radical prostatectomy: evolution in time and updated results. *Actas Urologicas Espanolas*.

[19] Katz R, Salomon L, Hoznek A, La Taille AD, Antiphon P, Claude Abbou C (2003). Positive surgical margins in laparoscopic radical prostatectomy: the impact of apical dissection, bladder neck remodeling and nerve preservation. *Journal of Urology*.

[20] Bollens R, Vanden Bossche M, Roumeguere T (2001). Extraperitoneal laparoscopic radical prostatectomy: results after 50 cases. *European Urology*.

[21] Rassweiler J, Sentker L, Seemann O, Hatzinger M, Rumpelt HJ (2001). Laparoscopic radical prostatectomy with the Heilbronn technique: an analysis of the first 180 cases. *Journal of Urology*.

[22] Guillonneau B, Vallancien G (2000). Laparoscopic radical prostatectomy: the Montsouris experience. *Journal of Urology*.

[23] Vickers AJ, Savage CJ, Hruza M (2009). The surgical learning curve for laparoscopic radical prostatectomy: a retrospective cohort study. *The Lancet Oncology*.

[24] Ramirez Backhaus M, Uwe Stolzenburg J, Do M, Dietel A, Ruiz-Cerdá JL, Jimenez Cruz JF (2009). Learning laparoscopic radical prostatectomy with the Leipzig program. Analysis of the training module program. *Actas Urologicas Espanolas*.

[25] Raboy A, Ferzli G, Albert P (1997). Initial experience with extraperitoneal endoscopic radical retropubic prostatectomy. *Urology*.

[26] Tobias-Machado M, Forseto P, Medina JA, Watanabe M, Juliano RV, Wroclawski ER (2004). Laparoscopic radical prostatectomy by extraperitoneal access with duplication of the open technique. *International Brazilian Journal of Urology*.

[27] Prasad SM, Maniar HS, Soper NJ, Damiano RJ, Klingensmith ME (2002). The effect of robotic assistance on learning curves for basic laparoscopic skills. *American Journal of Surgery*.

[28] Caballero Romeu JP, Palacios Ramos J, Pereira Arias JG, Gamarra Quintanilla M, Astobieta Odriozola A, Ibarluzea González G (2008). Radical prostatectomy: evaluation of learning curve outcomes laparoscopic and robotic-assisted laparoscopic techniques with radical retropubic prostatectomy. *Actas Urologicas Espanolas*.

